# Correction: Pattern-based sensing of aminoglycosides with fluorescent amphiphiles†
†Electronic supplementary information (ESI) available. See DOI: 10.1039/c4sc90043j


**DOI:** 10.1039/c4sc90043j

**Published:** 2014-10-24

**Authors:** Ziya Köstereli, Rosario Scopelliti, Kay Severin

**Affiliations:** a Institut des Sciences et Ingénierie Chimiques , Ecole Polytechnique Fédérale de Lausanne (EPFL) , 1015 Lausanne , Switzerland . Email: kay.severin@epfl.ch ; Fax: +41 21-693-9305

## Abstract

Correction for ‘Pattern-based sensing of aminoglycosides with fluorescent amphiphiles’ by Ziya Köstereli *et al.*, *Chem. Sci.*, 2014, **5**, 2456–2460.



## 

The publication and the ESI† describe the amphiphilic dye **2**. This compound should feature a C17 instead of a C18 alkane side chain.

Main text:

1) 1st page, right side, line 3 from the bottom, “octadecane” should be replaced by “heptadecane”.

2) Compound **2** depicted in Scheme 3 should feature a heptadecane and not an octadecane side chain.

ESI†:

1) The compounds **B** and **2** depicted in the reaction scheme should both feature a heptadecane and not an octadecane side chain.

The Royal Society of Chemistry apologises for these errors and any consequent inconvenience to authors and readers.

## Supplementary Material

Supplementary information

