# Supplementary material for: Correction: Pattern-based sensing of aminoglycosides with fluorescent amphiphiles
Source: Chem Sci. 2014 Oct 24;6(1):842. doi: 10.1039/c4sc90043j (PMC6063293; doi:10.1039/c4sc90043j)
Supplement: Supplementary file 1 [file SC-006-C4SC90043J-s001.pdf]

Electronic Supplementary Material (ESI) for Chemical Science.
This journal is © The Royal Society of Chemistry 2014

# Electronic Supplementary Information

## Pattern-Based Sensing of Aminoglycosides with Fluorescent Amphiphiles

Ziya Köstereli, Rosario Scopelliti and Kay Severin*

*Institut des Sciences et Ingénierie Chimiques,*
*École Polytechnique Fédérale de Lausanne (EPFL), Lausanne, Switzerland.*
*Fax: +41(0)21 6939305; Tel: +41(0)21 6939302; E-mail: kay.severin@epfl.ch*

**TABLE OF CONTENTS**

1. General S2
2. Syntheses S2
3. X-ray crystallography S9
4. UV-vis and fluorescence spectroscopy S11
5. Critical micelle concentrations S13
6. Transmission electron microscopy S15
7. Dynamic light scattering S16
8. Sensing studies S17
9. References S29

## 1. General:

All chemicals and solvents were purchased from standard suppliers and used without further purification. Stock solutions were prepared with bidistilled water and were stored at 4 °C. MOPS buffer (10 mM MOPS buffer, pH 7.0) was prepared by dissolving 3−(N−morpholino) propanesulfonic acid in bidistilled water. HCl and NaOH solutions were used to adjust the pH of the buffer. Fluorescence measurements were performed on a Varian Cary Eclipse fluorescence spectrophotometer at room temperature. Absorption spectra were measured on a Cary 50 bio spectrometer (Varian). Quartz cuvettes were used for the absorbance and fluorescence measurements. $^{1}$H and $^{13}$C NMR spectra were recorded on a Bruker Advance DPX 400 and 500 instruments at 25 °C. Multiplicities of the $^{1}$H NMR signals are assigned as following: s (singlet), d (doublet), t (triplet), dd (doublet of doublet), m (multiplet), (pp pseudo pentet). DLS measurements were performed with Zetasizer nano ZS90 (Malvern) instrument. High resolution mass spectra were recorded with a waters Q−TOF Ultima (ESI−TOF) instrument.

## 2. Syntheses:

### Synthesis of dye 1

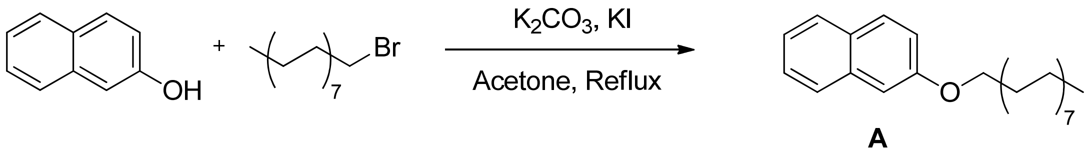


Compound **A** was synthesized in a similar fashion as described in the literature for a related compound:[1] 1-Bromohexadecane (5.28 g, 17.3 mmol), potassium carbonate (3.59, 26.0 mmol) and potassium iodide (~ 5 mg) were added to a refluxing solution of naphthalen-2-ol (2.50g, 17.3 mmol) in acetone (200 mL). The reaction mixture was heated under reflux with stirring for 24 h. The mixture was then cooled to 4 °C, resulting in the formation of a white precipitate. The precipitate was isolated by filtration and dissolved in chloroform (50 mL). After filtration of salts, the solvent was removed under reduced pressure to give compound **A** as a white solid (5.12 g, 13.9 mmol, 81%). Compound **A** was used for the synthesis of dye **1** without further purification.

$^{1}$H NMR (400 MHz, $CDCl_3$): $\delta$ = 0.81 (t, $J$ = 4.0 Hz, 3 H, $CH_3$), 1.17−1.34 (m, 24 H, $CH_2$), 1.43 (pp, $J$ = 6.6 Hz, 2 H, $CH_2$), 1.77 (pp, $J$ = 6.6 Hz, 2 H, $CH_2$), 4.00 (t, $J$ = 6.6 Hz, 2 H, $OCH_2$), 7.03−7.08 (m, 2 H, $CH_{arom}$), 7.22−7.27 (m, 1 H, $CH_{arom}$), 7.32−7.37 (m, 1 H, $CH_{arom}$), 7.62−7.69 (m, 3 H, $CH_{arom}$). $^{13}$C NMR (100 MHz, $CDCl_3$): $\delta$ = 14.12, 22.70, 26.13, 29.27, 29.37, 29.44, 29.61, 29.62, 29.68, 29.71, 31.94, 68.05, 106.57, 119.03, 123.43, 126.25, 126.67, 127.62, 128.88, 129.28, 134.63, 157.12. ESI−MS calcd. for $C_{26}H_{40}O$ [(M)] m/z = 369.3157 found 369.3168.

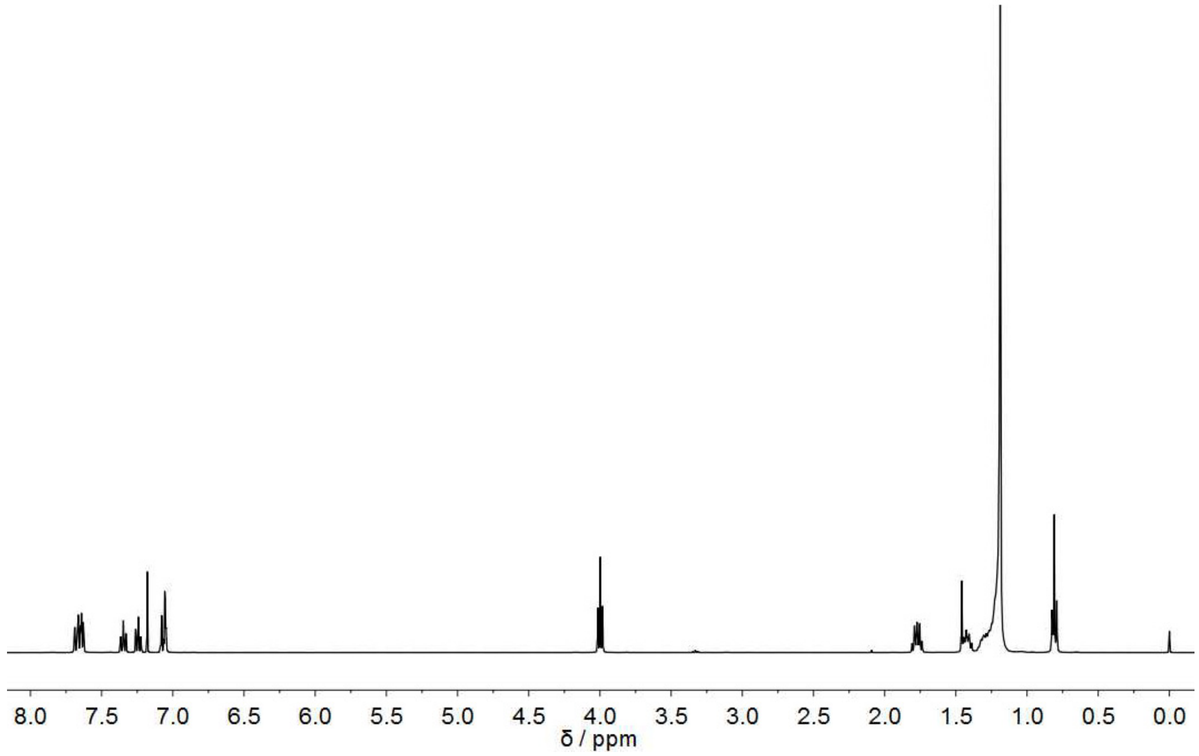


**Figure S1.** $^{1}$H NMR spectrum of compound **A** (400 MHz, $CDCl_3$).

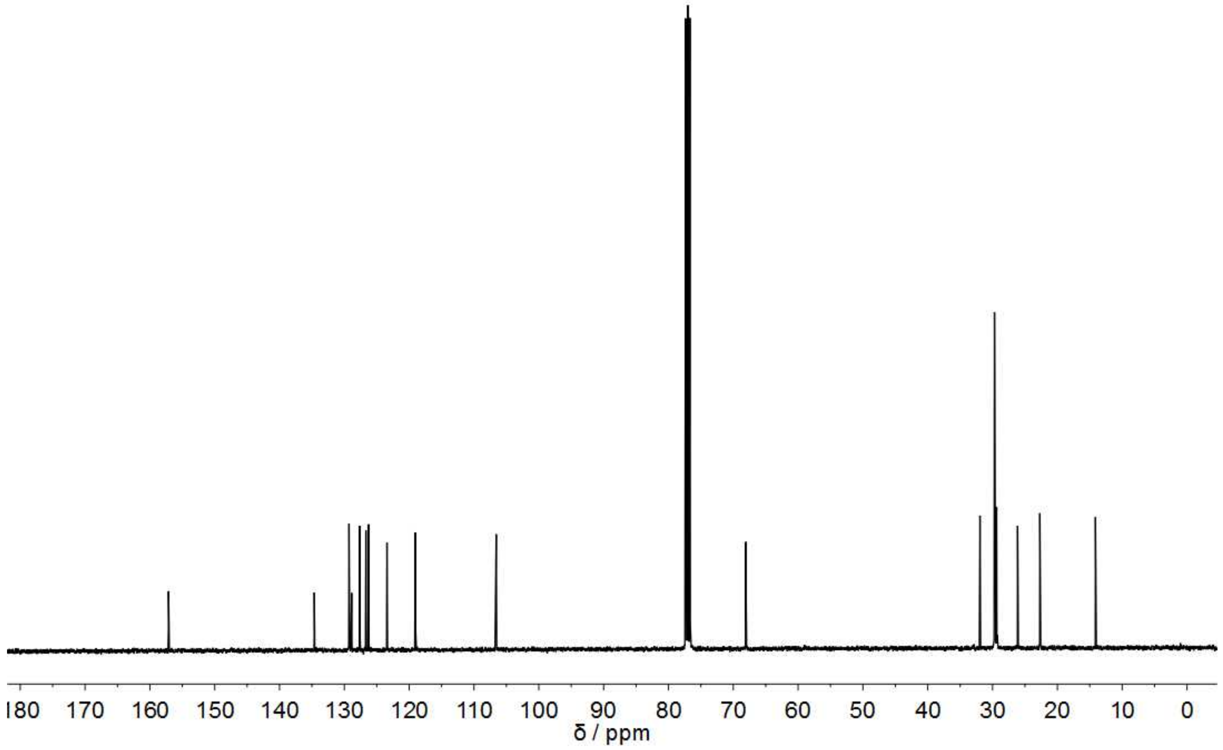


**Figure S2.** $^{13}$C NMR spectrum of compound **A** (100 MHz, $CDCl_3$).

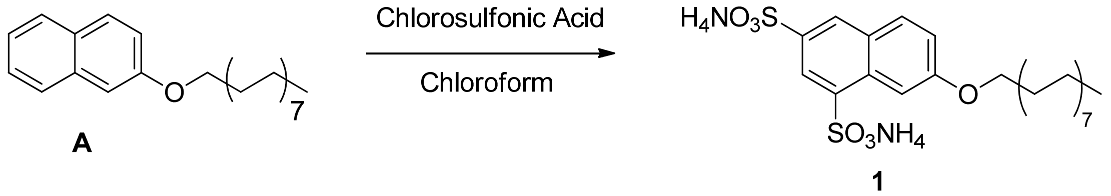


Dye **2** was synthesized in a similar fashion as described in the literature for a related compound:[2] A solution of chlorosulfonic acid (3.44 mL, 51.8 mmol) in $CHCl_3$ (40 mL) was added dropwise over 1 h under stirring to a cooled (0 °C) solution of compound **A** (1.73 g, 4.71 mmol) in $CHCl_3$ (20 mL). The ice bath was then removed and the mixture was stirred for additional 2 h at RT resulting in the formation of a white precipitate. The precipitate was isolated by filtration, washed with chloroform, and redissolved in water (~ 50 mL). The pH of the solution was adjusted to pH 7 by addition of aqueous NaOH (8 M), and the solution was dried under vacuum. Purification by column chromatography ($SiO_2$; eluent: ammonia (25 %):isopropanol; 1:2) gave dye **2**, which still contained minor amounts of a second isomer.

After recrystallization from methanol/hexane for three times and drying in vacuum, dye **1** was obtained as pure product (0.61 g, 1.08 mmol, 23%).

$^1$H NMR (400 MHz, $CD_3OD$): $\delta$ = 0.92 (t, $J$ = 6.3 Hz, 3 H, $CH_3$), 1.28−1.50 (m, 24 H, $CH_2$), 1.56 (pp, $J$ = 6.6 Hz, 2 H, $CH_2$), 1.88 (pp, $J$ = 6.6 Hz, 2 H, $CH_2$), 4.21 (t, $J$ = 6.4 Hz, 2 H, $OCH_2$), 7.24 (dd, $J$ = 9.0, 2.5 Hz, 1 H, $CH_{arom}$), 7.90 (d, $J$ = 9.0 Hz, 1 H, $CH_{arom}$), 8.26 (d, $J$ = 2.5 Hz, 1 H, $CH_{arom}$), 8.35 (d, $J$ = 1.9 Hz, 1 H, $CH_{arom}$), 8.62 (d, $J$ = 1.9 Hz, 1 H, $CH_{arom}$). $^{13}$C NMR (100 MHz, $CD_3OD$): $\delta$ = 14.44, 23.74, 27.27, 30.38, 30.48, 30.58, 30.76, 30.80, 33.08, 69.21, 107.18, 121.04, 124.77, 129.24, 130.00, 131.59, 132.53, 139.86, 141.12, 160.20. ESI−MS calcd. for $C_{26}H_{38}S_2O_7Na_2$ [(M-2$NH_4$+2Na)] m/z = 573.1932 found 573.1946.

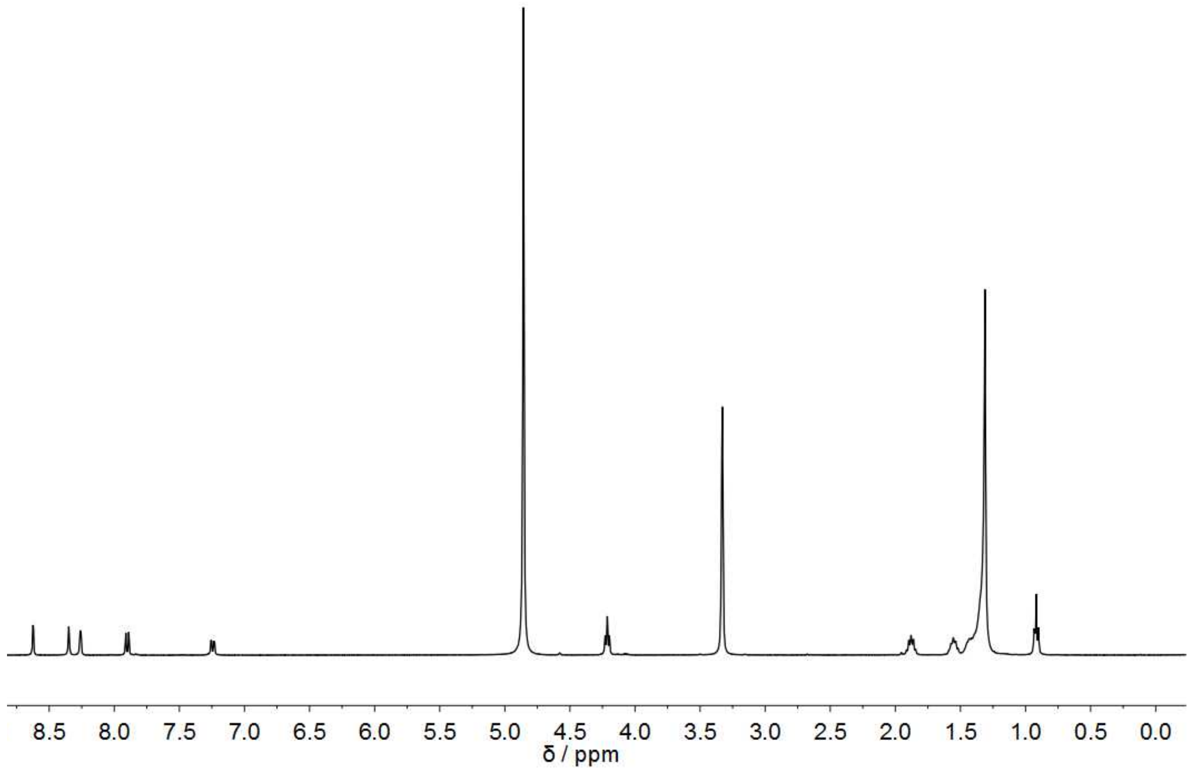


**Figure S3.** $^1$H NMR spectrum of dye **1** (400 MHz, $CD_3OD$).

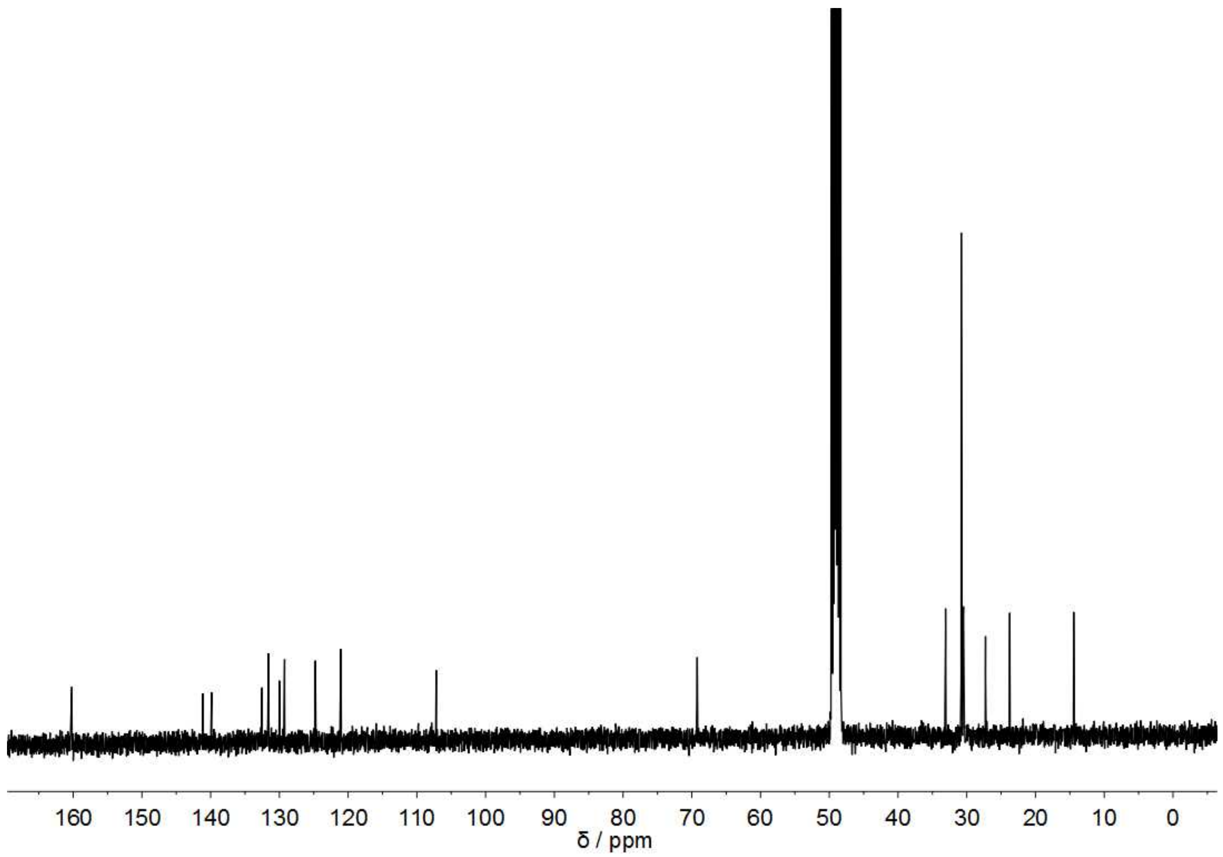


**Figure S4.** $^{13}$C NMR spectrum of dye **1** (100 MHz, $CD_3OD$).

**Synthesis of dye 2**

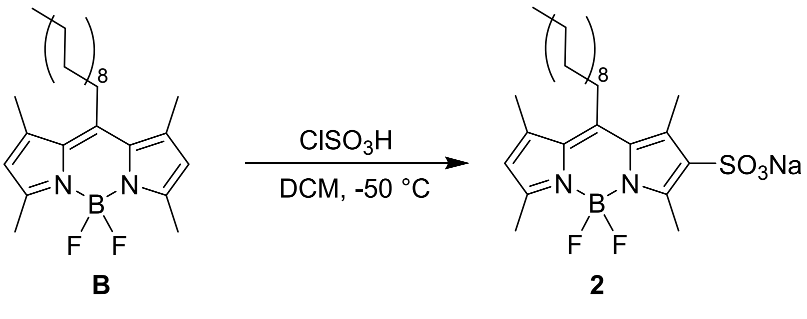


Compound **B** and dye **2** were synthesized in a similar fashion as described in the literature:[3] A solution of chlorosulfonic acid (26.6 µL, 0.4 mmol) in $CH_2Cl_2$ (5 mL) was added dropwise over 20 min under stirring to a cooled (−50 °C) solution of compound **B** (100 mg, 0.20 mmol) in $CH_2Cl_2$ (20 mL). The ice bath was then removed and the mixture was stirred to reach RT resulting in the formation of a red precipitate. The precipitate was isolated by filtration, washed with $CH_2Cl_2$, and redissolved in aqueous bicarbonate solution (10 mL, 40 mM). The

solution was dried under vacuum. Purification by column chromatography ($SiO_2$; eluent: $SiO_2$; eluent: $CHCl_3$:MeOH:$H_2O$; 7:3:0.5) gave **2** as a red solid (28 mg, 0.046 mmol, 24%).

$^1$H NMR (400 MHz, $CD_3OD$): $\delta$ = 0.80 (t, $J$ = 7.0 Hz, 3 H, $CH_3$), 1.15−1.35 (m, 28 H, $CH_2$), 1.44 (p, $J$ = 7.5 Hz, 2 H, $CH_2$), 1.50−1.65 (m, 2 H, $CH_2$), 2.38 (s, 3 H, $CH_3$), 2.39 (s, 3 H, $CH_3$), 2.62 (s, 3 H, $CH_3$), 2.64 (s, 3 H, $CH_3$), 2.98−3.04 (m, 2 H, $CH_2$), 6.14 (s, 1 H, $CH_{arom}$). $^{13}$C NMR (125 MHz, $CD_3OD$): $\delta$ = 12.61, 12.92, 13.12, 15.45, 22.24, 28.02, 28.97, 29.26, 29.79, 31.50, 31.58, 122.58, 129.17, 132.59, 132.84, 137.30, 142.93, 148.61, 150.60, 156.38. ESI−MS calcd. for $C_{30}H_{49}BF_2N_2O_3S$ [(M-Na+H)] m/z = 565.3452 found 565.3448.

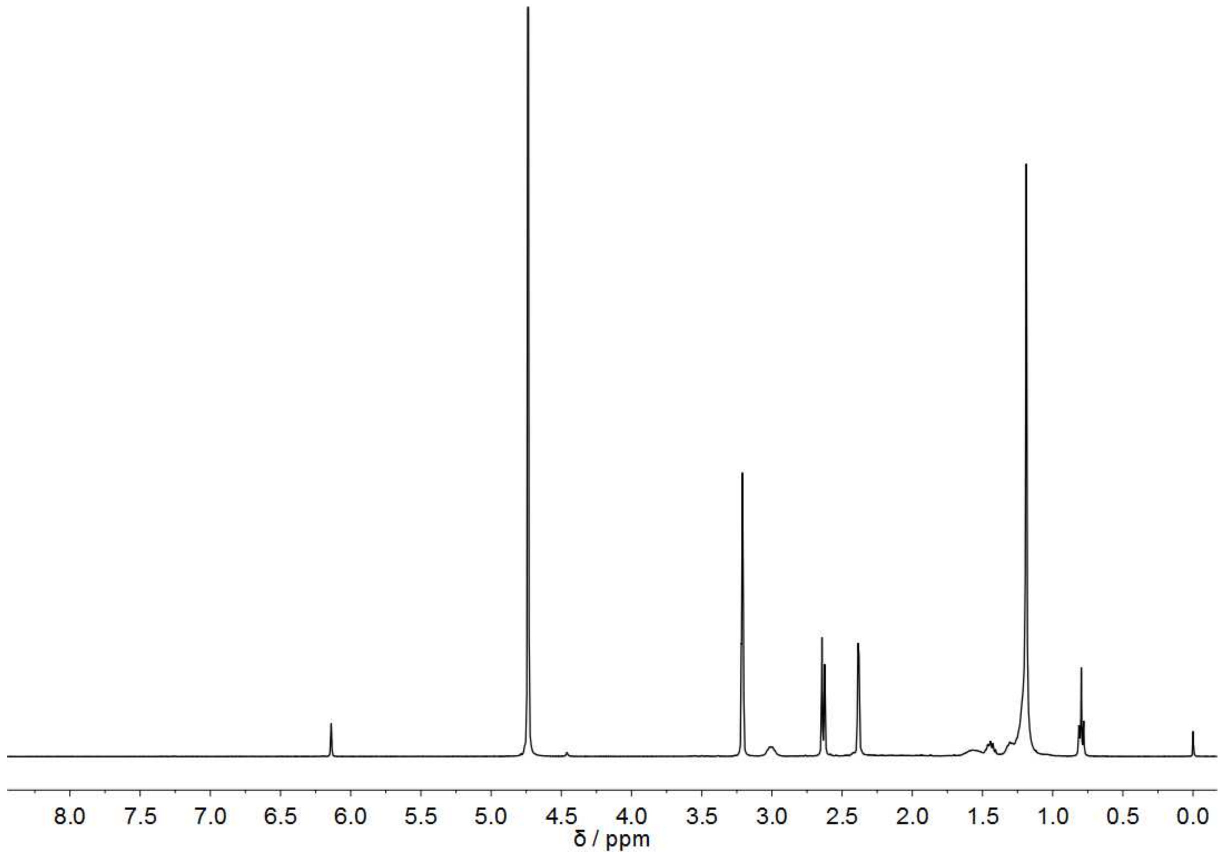


**Figure S5.** $^1$H NMR spectrum of dye **2** (400 MHz, $CD_3OD$).

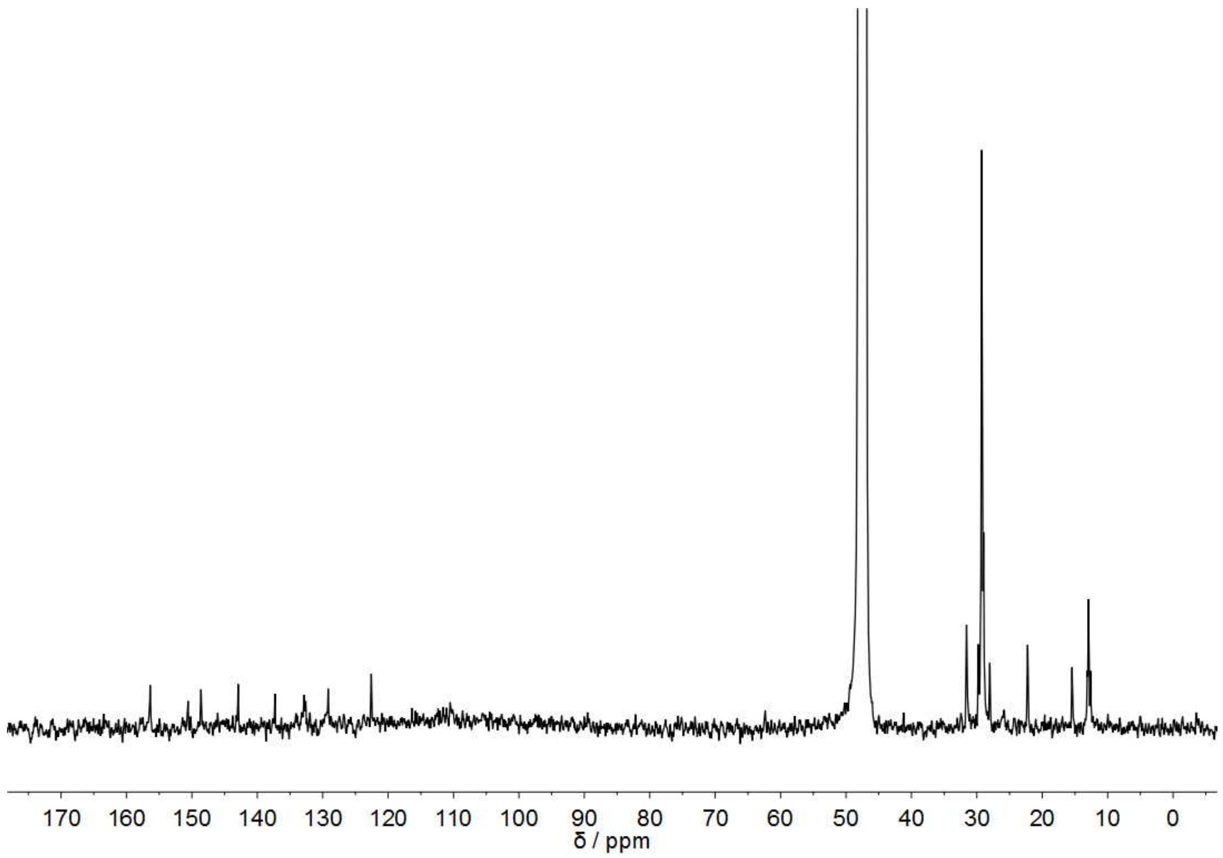


**Figure S6.** $^{13}C$ NMR spectrum of dye **2** (125 MHz, $CD_3OD$). The quality of the spectrum is not very good because of the poor solubility of **2**.

## 3. X-ray crystallography:

Single crystals of **1** were obtained by liquid-liquid diffusion in a test tube (uper layer: hexane, low layer: methanol). The data collection of compound **1** was measured at room temperature using Cu $K_\alpha$ radiation on an Agilent Technologies SuperNova dual system in combination with an Atlas CCD detector. Data reduction was carried out with CrysAlis PRO.[4] The solution and refinement were performed by SHELX.[5] The crystal structure was refined using full-matrix least-squares based on $F^2$ with all non hydrogen atoms anisotropically defined. Hydrogen atoms were placed in calculated positions by means of the "riding" model. In the final stages of the refinement, some restraints (SHELX cards: SIMU and SADI) were applied to disordered solvent ($CH_3OH$) and to the geometry of cations ($NH_4^+$). The crystallographic data have been deposited at the Cambridge Crystallographic Data Center (CCDC 991057).

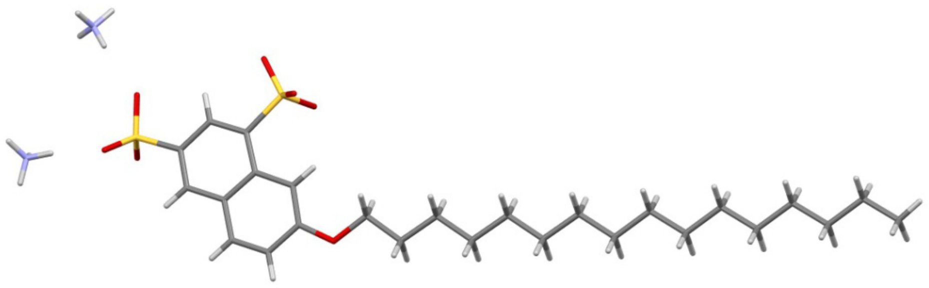

**Figure S7.** Molecular structure of dye **1** in the crystal.

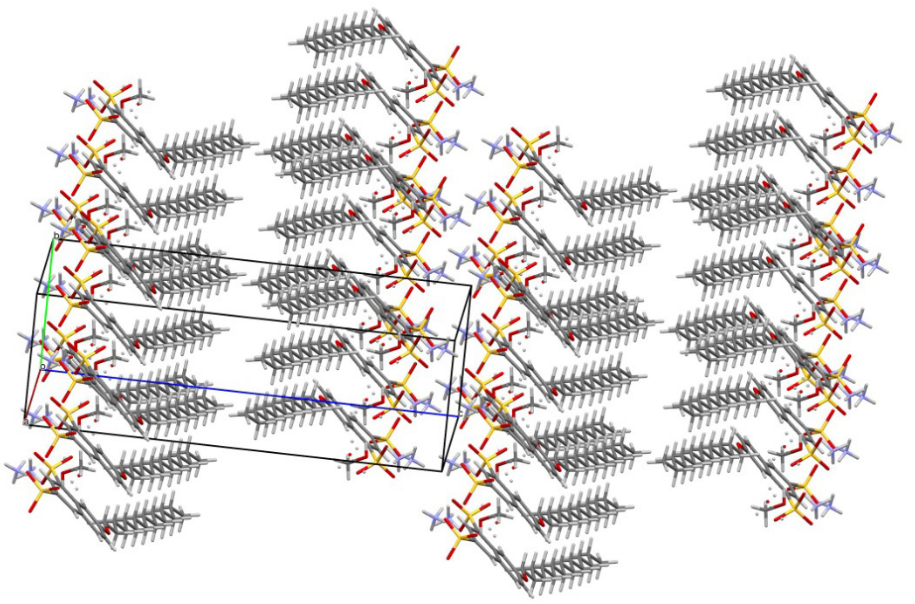

**Figure S8.** Packing of dye **1** in the crystal.

Table 1. Crystal data and structure refinement for dye **1**.

| | | |
|---|---|---|
| Identification code | Dye 1 | |
| Empirical formula | $C_{27}H_{50}N_2O_8S_2$ | |
| Formula weight | 594.81 | |
| Temperature | 293(2) K | |
| Wavelength | 1.54178 Å | |
| Crystal system | Triclinic | |
| Space group | *P*-1 | |
| Unit cell dimensions | a = 6.53547(18) Å | α= 83.956(3)°. |
| | b = 9.4651(4) Å | β= 84.790(3)°. |
| | c = 26.1930(10) Å | γ = 89.716(3)°. |
| Volume | 1604.59(10) $Å^3$ | |
| *Z* | 2 | |
| Density (calculated) | 1.231 $Mg/m^3$ | |
| Absorption coefficient | 1.892 $mm^{-1}$ | |
| F(000) | 644 | |
| Crystal size | 0.49 x 0.10 x 0.06 $mm^3$ | |
| Theta range for data collection | 3.41 to 73.28°. | |
| Index ranges | -4<=h<=7, -11<=k<=11, -30<=l<=32 | |
| Reflections collected | 10534 | |
| Independent reflections | 6205 [*R*(int) = 0.0164] | |
| Completeness to theta = 67.00° | 99.6 % | |
| Absorption correction | Analytical | |
| Max. and min. transmission | 0.909 and 0.568 | |
| Refinement method | Full-matrix least-squares on $F^2$ | |
| Data / restraints / parameters | 6205 / 79 / 396 | |
| Goodness-of-fit on $F^2$ | 1.037 | |
| Final R indices [I>2sigma(I)] | $R_1$ = 0.0335, w$R_2$ = 0.0935 | |
| *R* indices (all data) | $R_1$ = 0.0375, w$R_2$ = 0.0980 | |
| Extinction coefficient | 0.0012(2) | |
| Largest diff. peak and hole | 0.408 and -0.349 e.$Å^{-3}$ | |

## 4. UV-vis and fluorescence spectroscopy:

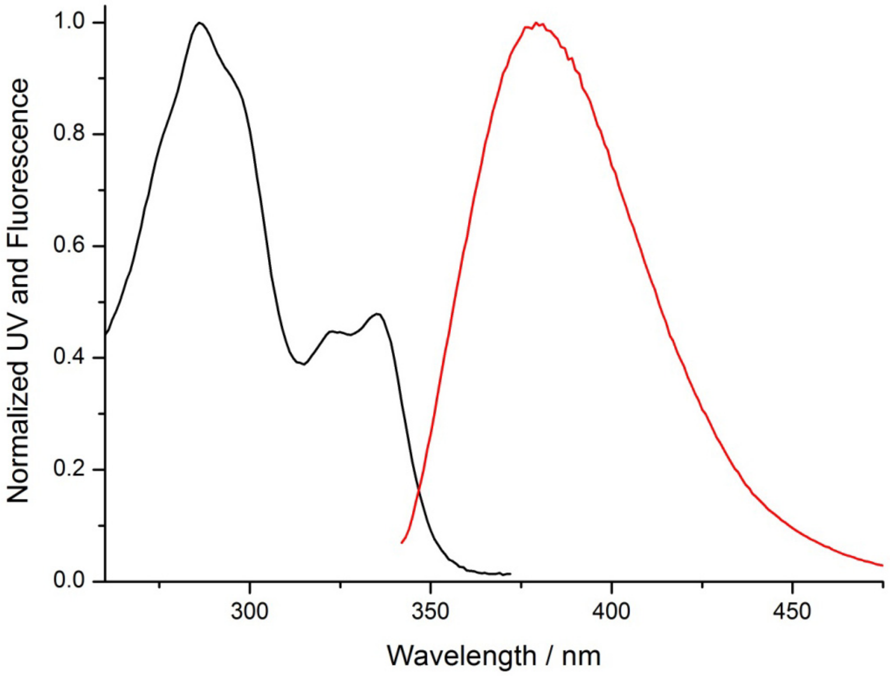


**Figure S9.** Normalized UV-vis (black) and fluorescence (red) spectra ($\lambda_{ex}$: 335 nm) of buffered aqueous solutions (10 mM MOPS, pH 7.0) containing dye **1** (10 μM).

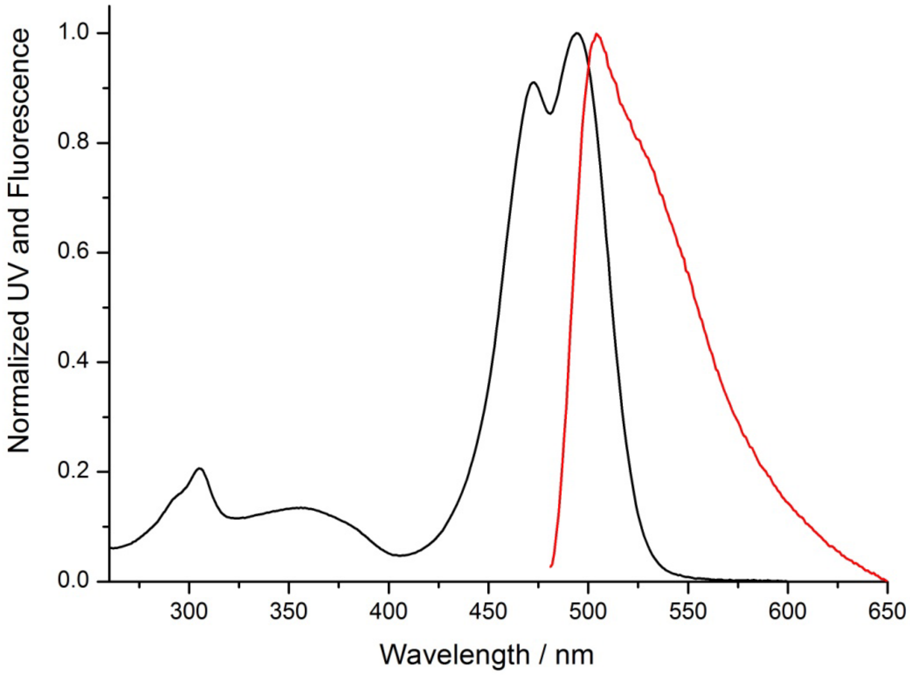


**Figure S10.** Normalized UV-vis (black) and fluorescence (red) spectra ($\lambda_{ex}$: 470 nm) of buffered aqueous solutions (10 mM MOPS, pH 7.0) containing dye **2** (10 μM).

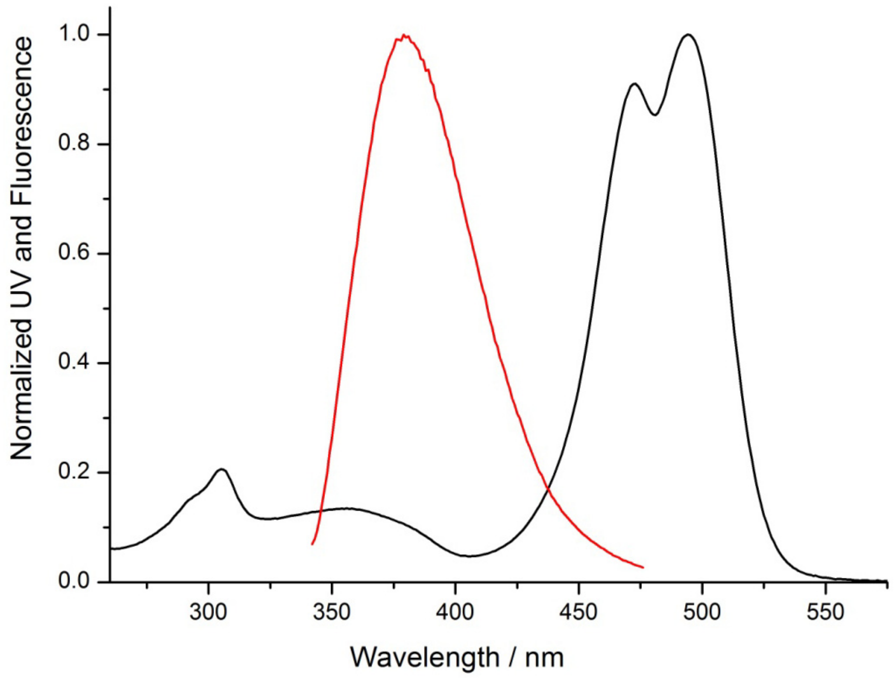


**Figure S11.** Normalized UV-vis spectrum of a buffered aqueous solution (10 mM MOPS, pH 7.0) containing dye **2** (black line, [**2**] = 10 µM), and normalized fluorescence spectrum of a solution containing dye **1** (red line, [**1**] = 10 µM, $\lambda_{ex}$: 335 nm).

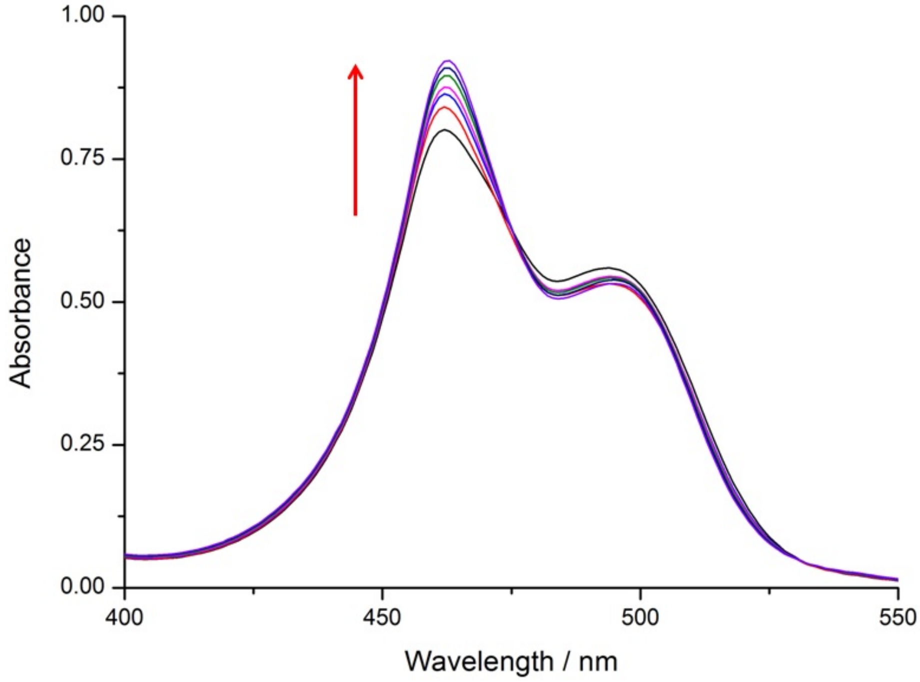


**Figure S12.** UV-vis spectra of buffered aqueous solutions (10 mM MOPS, pH 7.0) containing dye **2** (30 µM) and different amounts of dye **1** (0–30 µM). The red arrow indicates the changes with increasing concentration of dye **1**.

## 5. Critical micelle concentrations:

Stock solutions of dye **1** (2.0 mM) and **2** (50 μM) were prepared in 10 mM MOPS buffer (pH 7.0). For the fluorescence measurements, aliquots of the stock solutions were diluted with MOPS buffer (10 mM MOPS, pH 7.0). The fluorescence spectra of the resulting solutions were recorded at RT ($\lambda_{ex}$: 335 nm for **1**, $\lambda_{ex}$: 460 nm for **2**). The fluorescence emission maxima of solutions of dye **1** shift from 378 to 390 nm upon increasing the concentration from 1 to 256 μM (Fig. S13, left side). Similarly, the fluorescence emission maxima for solutions of **2** shift from 503 to 529 nm upon increasing the concentrations from 1 to 45 μM (Fig. S13, right side). The cmc was determined by linear extrapolation as described in the literature.[6]

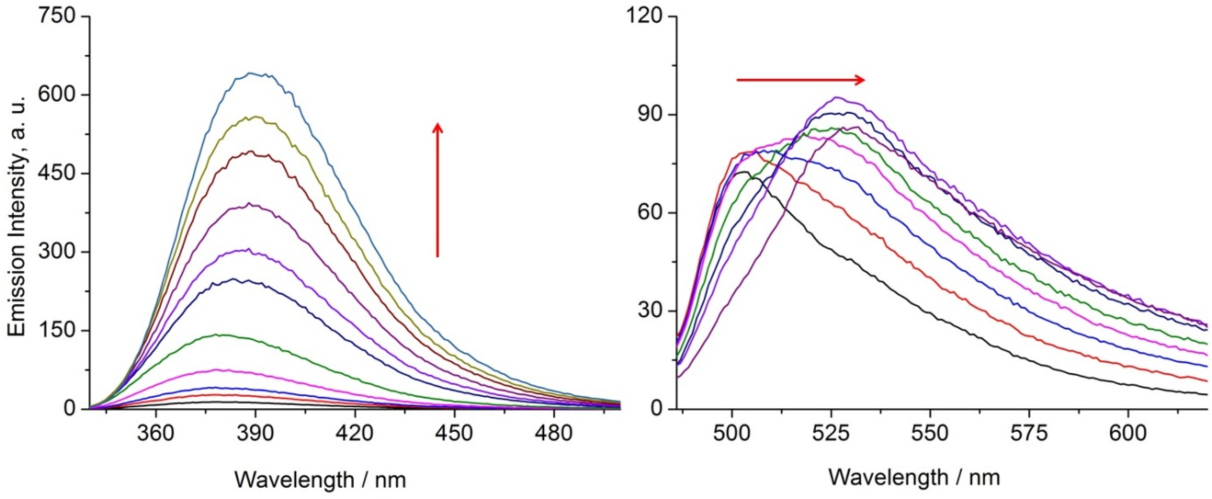


**Figure S13.** Fluorescence emission spectra of buffered aqueous solutions (10 mM MOPS, pH 7.0) containing dye **1** (left, $\lambda_{ex}$: 335 nm, 1−256 μM) and **2** (right, $\lambda_{ex}$: 460 nm, 1−45 μM). The red arrows indicate the changes with increasing concentration.

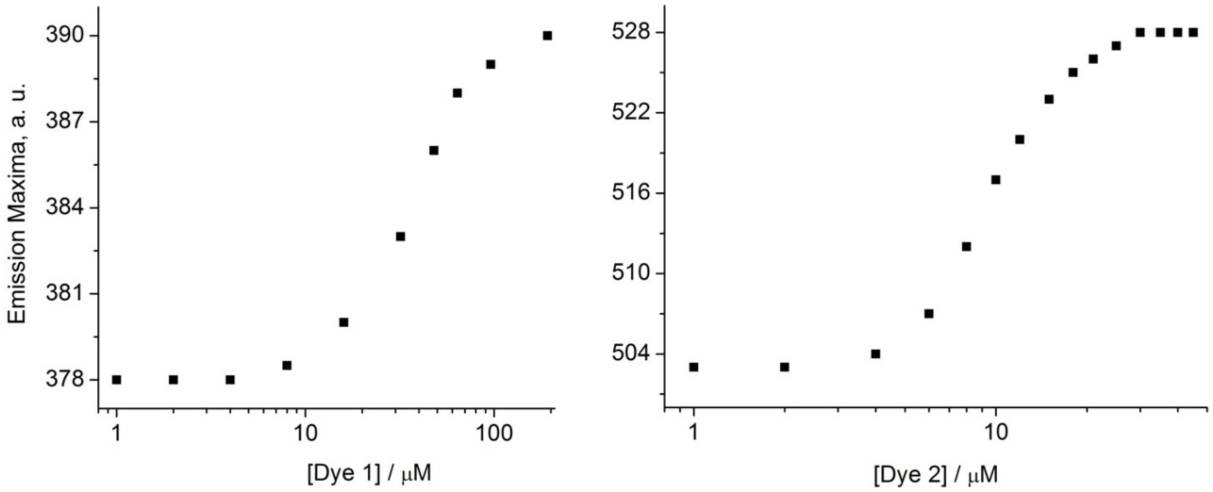


**Figure S14.** Fluorescence emission maxima of buffered aqueous solutions (10 mM MOPS, pH 7.0) containing increasing amounts of dye **1** ($\lambda_{ex}$: 335 nm, 1−512 μM) or **2** ($\lambda_{ex}$: 460 nm, 1−45 μM). The graphs are plotted on a logarithmic scale.

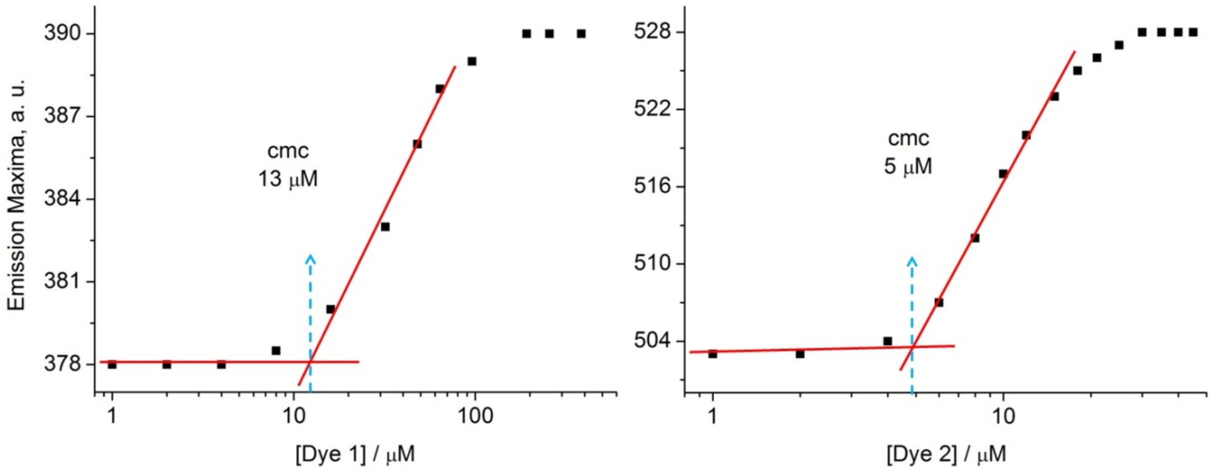


**Figure S15.** Critical micelle concentration (cmc) of dye **1** and **2**. The graphs are plotted on a logarithmic scale.

## 6. Transmission electron microscopy:

Two samples were prepared for TEM measurement. A solution of dye **2** (2.4 mM, 10 mM MOPS, pH 7.0) was measured as the first sample (Figure S15). For the second sample dye **2** (2.4 mM, 10 mM MOPS, pH 7.0) and neomycin (2.4 mM, 10 mM MOPS, pH 7.0) were mixed in a vial then filtered (PTFE filter, 0.22 μM). The resulting solution was used for the measurement. Samples were placed on a Lacey carbon coated 300 mesh copper grid. Uranyl acetate solution (2 wt%) was used for staining. A drop of the respective solution (20 μL) was put on a parafilm sheet. The grid was placed on the top of the drop with its upper side down and kept there for 2 min. Then, an aliquot of the uranyl acetate solution (20 μL, 2 wt%) was put on a other parafilm sheet. After 2 minutes the grid was taken and placed on the top of the drop with its upper side down and kept there for 5 min. Stained grids were dried in the fume hood at room temperature.

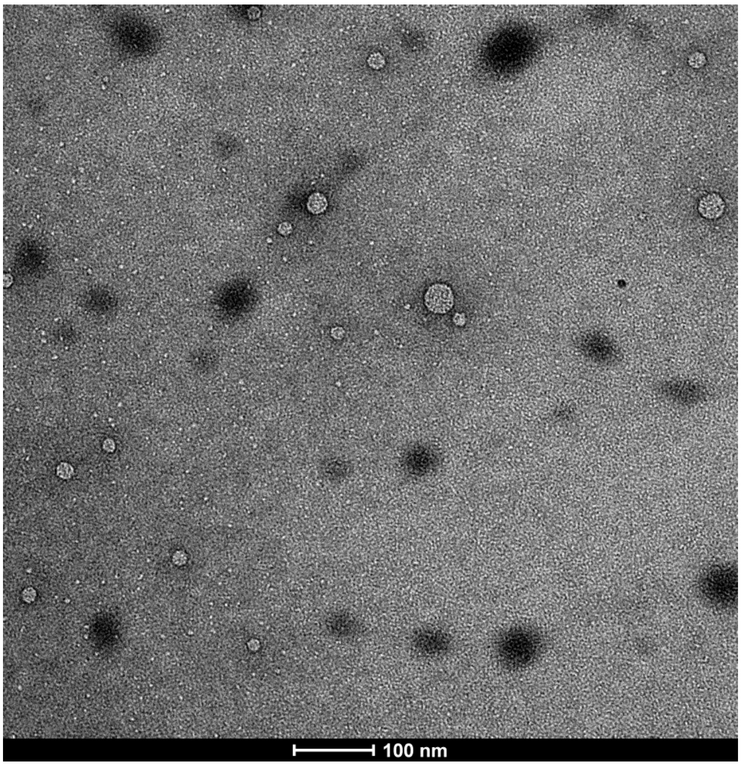


**Figure S16.** TEM image of a sample containing dye **1** (for sample preparation see text above). The image reveals inhomogeneous spherical aggregates with the size between 8−30 nm (scale bar = 100 nm). The black spots are due to accumulation of the staining agent.

## 7. Dynamic light scattering:

Two samples were prepared for DLS measurements. Buffered aqueous solutions of dye **1** (1.0 mM) and dye **2** (50 μM) were prepared (10 mM MOPS, pH 7.0) and then filtered (PTFE filter, 0.22 μM). The solutions were found to contain polydisperse aggregates with an average size of 6 nm (**1**) and 12 nm (**2**), respectively.

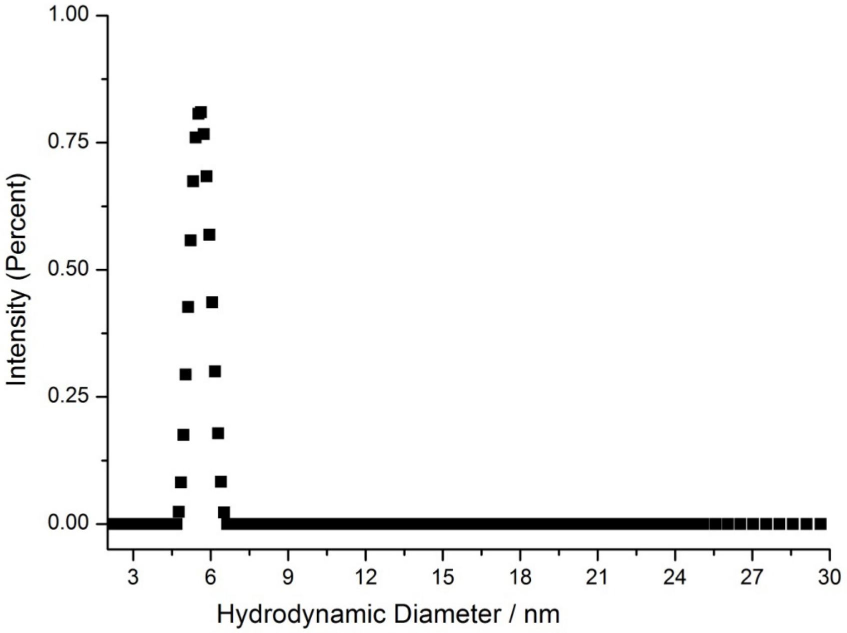


**Figure S17.** Typical DLS size distribution for solutions of dye **1** (1.0 mM)

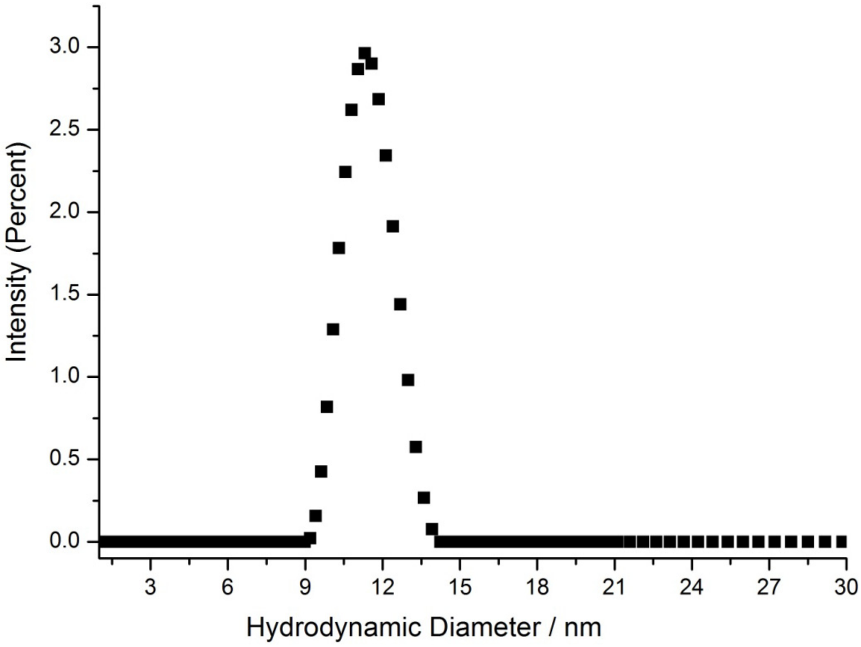


**Figure S18.** Typical DLS size distribution for solutions of dye **2** (50 μM).

## 8. Sensing studies:

### Fluorescence sensing of aminoglycosides

Stock solutions of dye **1** (2.0 mM), dye **2** (50 μM), and the analytes (2.0 mM) were prepared in 10 mM MOPS buffer (pH 7.0). For the measurements, the stock solutions were diluted with buffered water (10 mM MOPS, pH 7.0). The final volume in all samples was 1.5 mL. The fluorescent signal was measured 10 min after sample preparation. A mixture of dye **1** and **2** ($[\mathbf{1}]_{final}$ = 4 μM; $[\mathbf{2}]_{final}$ = 30 μM) in buffered aqueous solutions (10 mM MOPS, pH 7.0) was used as the sensing system. Seven different analytes (apramycin, paromomycin, neomycin, amikacin, kanamycin B, kanamycin A, and gentamicin) were employed. These analytes were also tested with dye **1** (4 μM) and dye **2** (5 and 30 μM) individually. The three aminoglycosides apramycin (A), paromomycin (P), and kanamycin B (KB), as well as equimolar mixtures of A+KB, A+P, P+KB and A+P+KB (total conc. = 10 μM) were selected for selectivity test.

### Multivariate analysis

Five independent fluorescence measurements were performed for each sample. The data were analyzed with the help of the statistics software SYSTAT 11. A stepwise variable selection algorithm was used to select appropriate wavelength for the multivariate analyses. Fluorescence emission values in the region between 365 and 620 nm with incremental steps of 5 nm (365, 370, 375, etc) were used as input for the variable selection algorithm. Four wavelengths were selected: 380 nm, 515 nm, 545 nm, and 615 nm. The emission values at these four wavelengths were used as input for linear discriminant analyses (LDA) and principal component analyses (PCA).

## Sensing studies with dye mixtures

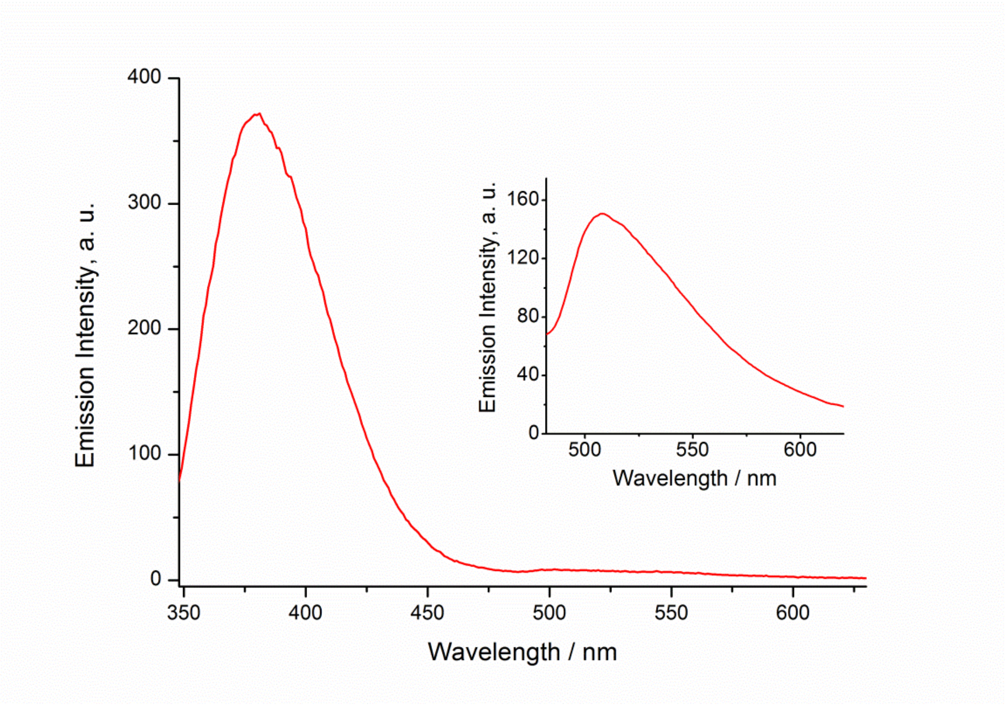


**Figure S19.** Fluorescence emission spectra ($\lambda_{ex}$: 335 nm) of solutions containing a mixture of dye **1** and **2** ([**1**] = 4 µM; [**2**] = 30 µM) in MOPS buffer (10 mM, pH 7.0). The inset shows the fluorescence emission of dye **2**. This spectrum was obtained using a slightly larger slit width on the spectrometer.

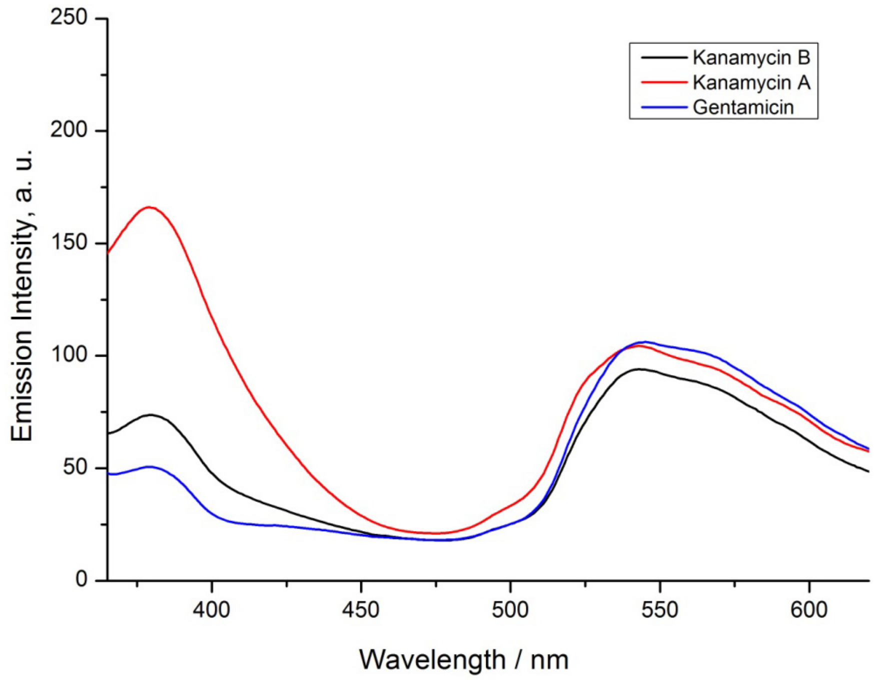


**Figure S20.** Fluorescence emission spectra ($\lambda_{ex}$: 335 nm) of solutions containing a mixture of dye **1** and **2** ([**1**] = 4 µM; [**2**] = 30 µM) and the analytes gentamicin (blue line), kanamycin A (red line), or kanamycin B (black line) ([analyte] = 10 µM; 10 mM MOPS, pH 7.0).

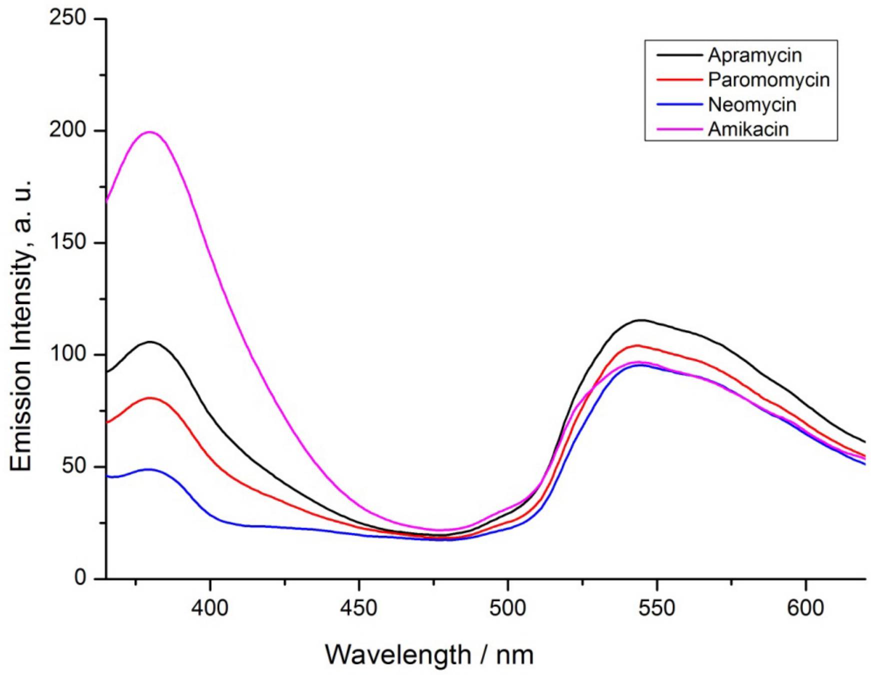


**Figure S21.** Fluorescence emission spectra ($\lambda_{ex}$: 335 nm) of solutions containing a mixture of dye **1** and **2** ([**1**] = 4 μM; [**2**] = 30 μM) and the analytes neomycin (blue line), paromomycin (red line), apramycin (black line), or amikacin (pink line) ([analyte] = 10 μM; 10 mM MOPS, pH 7.0).

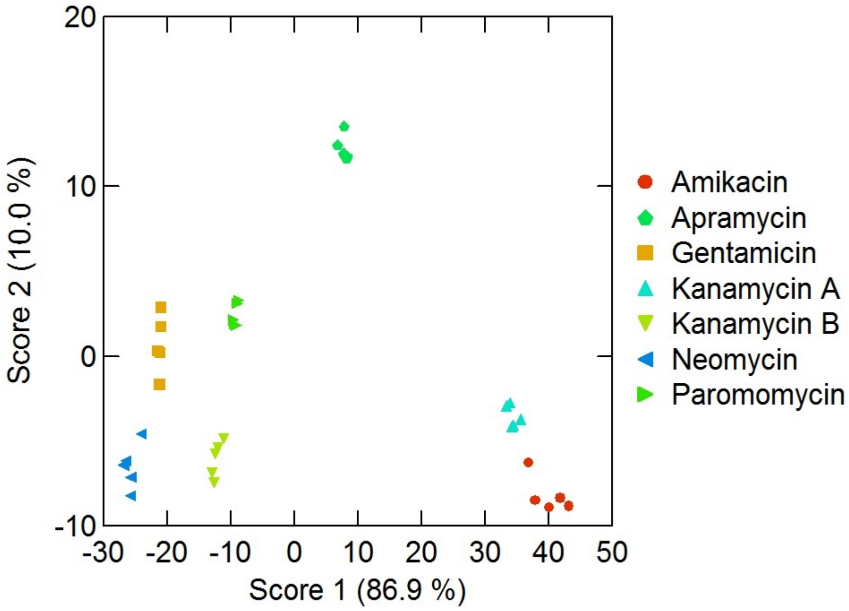


**Figure S22.** Two-dimensional LDA score plot for the analysis of seven different aminoglycosides. The data were obtained as described above.

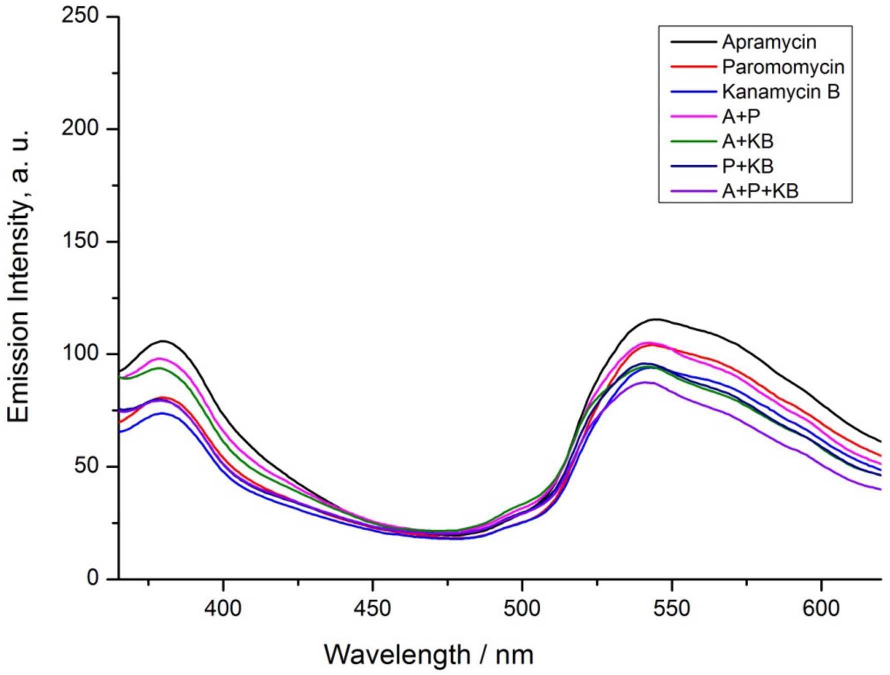


**Figure S23.** Fluorescence emission spectra ($\lambda_{ex}$: 335 nm) of solutions containing a mixture of dye **1** and **2** ([**1**] = 4 µM; [**2**] = 30 µM) and the analytes apramycin (A), paromomycin (P), kanamycin B (KB), or equimolar mixtures of these aminoglycosides ($[analyte]_{total}$ = 10 µM; 10 mM MOPS, pH 7.0).

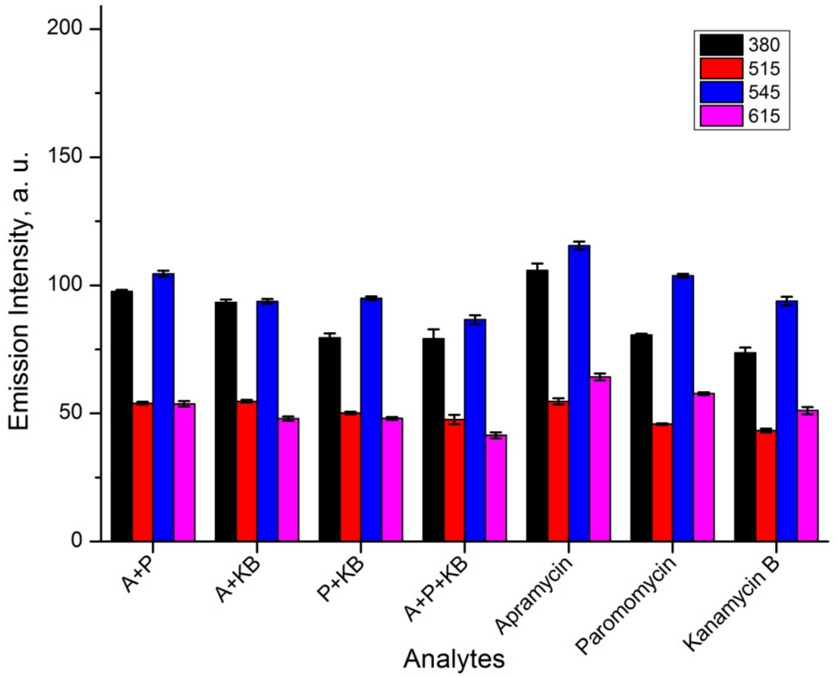


**Figure S24.** Fluorescence emission intensities ($\lambda_{ex}$: 335 nm) at 380 nm (black), 515 nm (red), 545 nm (blue), and 615 nm (pink) of solutions containing a mixture of dye **1** and **2** ([**1**] = 4 µM; [**2**] = 30 µM) and the analytes apramycin (A), paromomycin (P), kanamycin B (KB), or equimolar mixtures of these aminoglycosides ($[analyte]_{total}$ = 10 µM; 10 mM MOPS, pH 7.0). The values represent averages of five independent measurments.

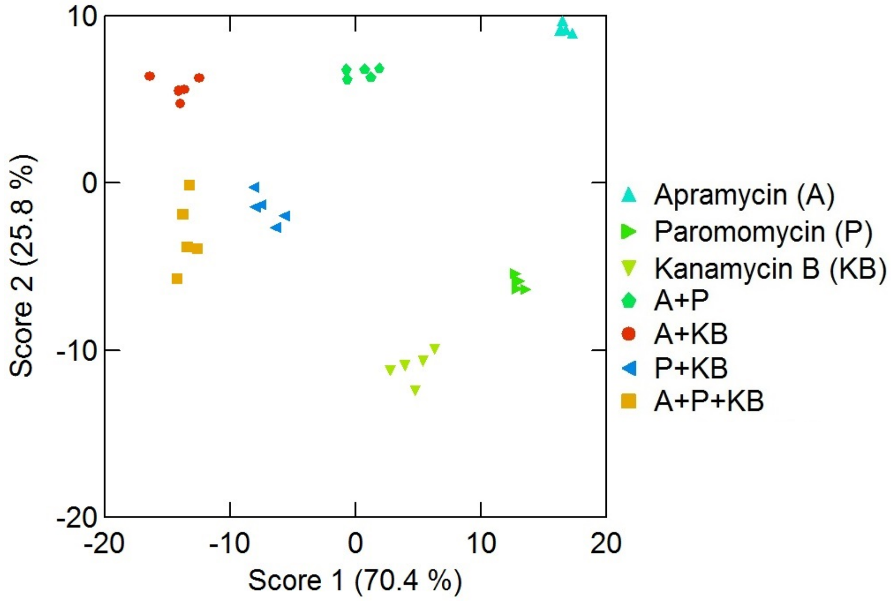


**Figure S25.** Two-dimensional LDA score plot for the analysis of apramycin (A), paromomycin (P), kanamycin B (KB), and equimolar mixtures of these aminoglycosides. The data were obtained as described above.

## Sensing studies with dye 1 alone

The solutions were prepared as described above. A stepwise variable selection algorithm was employed to select three wavelengths: 375, 390, and 410 nm. The emission values at these wavelengths were used as input for linear discriminant analyses (LDA) and principal component analyses (PCA).

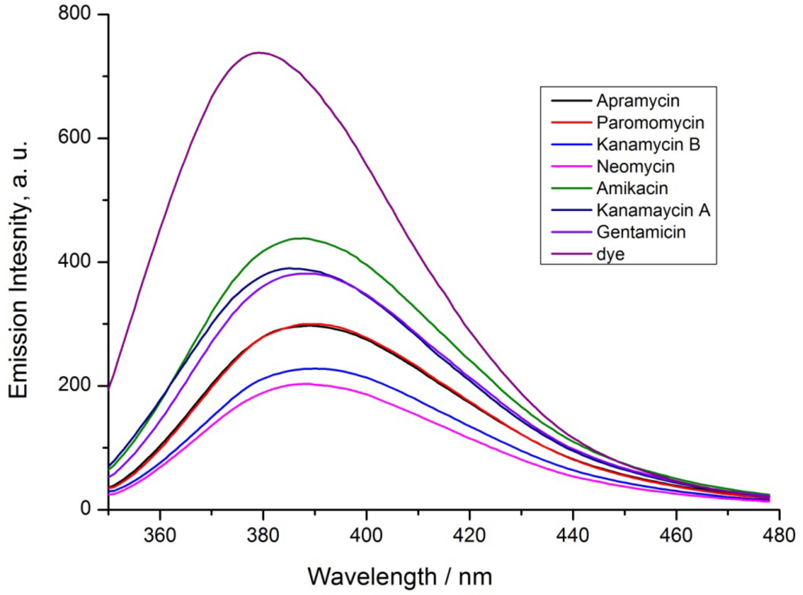


**Figure S26.** Fluorescence emission spectra ($\lambda_{ex}$: 335 nm) of solutions containing dye **1** (4 µM) and the analytes paromomycin, kanamycin B, kanamycin A, amikacin, neomycin, gentamicin, or apramycin ([analyte] = 10 µM; 10 mM MOPS, pH 7.0).

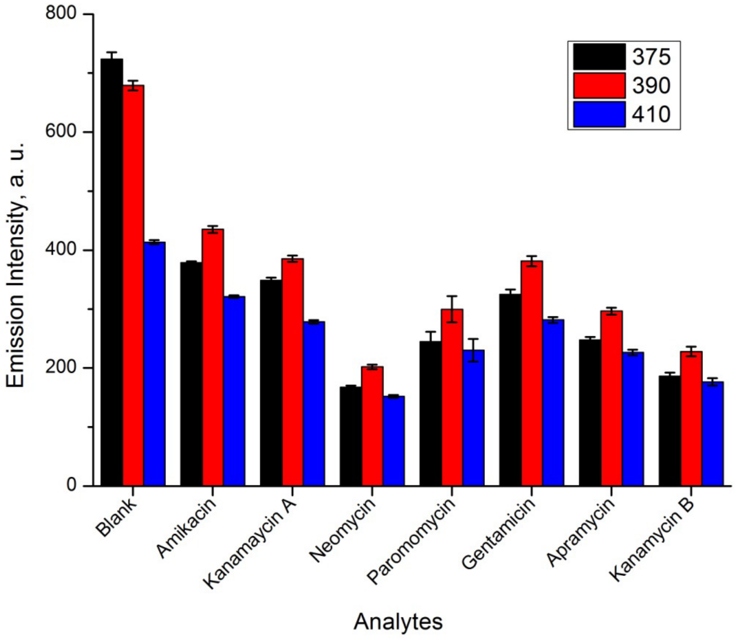


**Figure S27.** Fluorescence emission intensities ($\lambda_{ex}$: 335 nm) at 375 nm (black), 390 nm (red), and 410 nm (blue) of solutions containing dye **1** (4 µM) and the analytes paromomycin, kanamycin B, kanamycin A, amikacin, neomycin, gentamicin, or apramycin ([analyte] = 10 µM; 10 mM MOPS, pH 7.0). The values represent averages of five independent measurments.

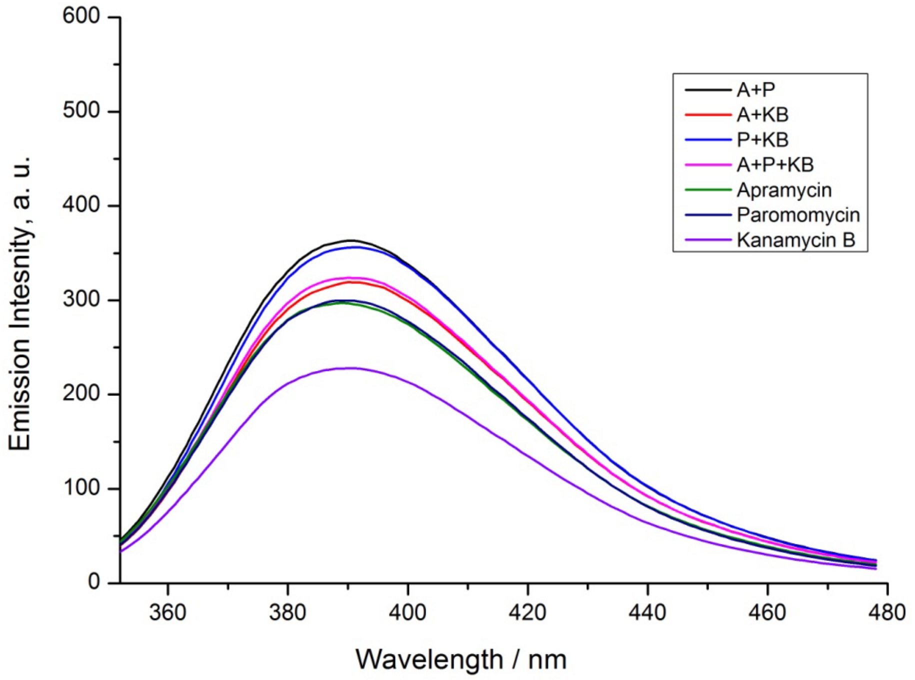


**Figure S28.** Fluorescence emission spectra ($\lambda_{ex}$: 335 nm) of solutions containing dye **1** (4 µM) and the analytes apramycin (A), paromomycin (P), kanamycin B (KB), or equimolar mixtures of these aminoglycosides ([analyte]$_{total}$ = 10 µM; 10 mM MOPS, pH 7.0).

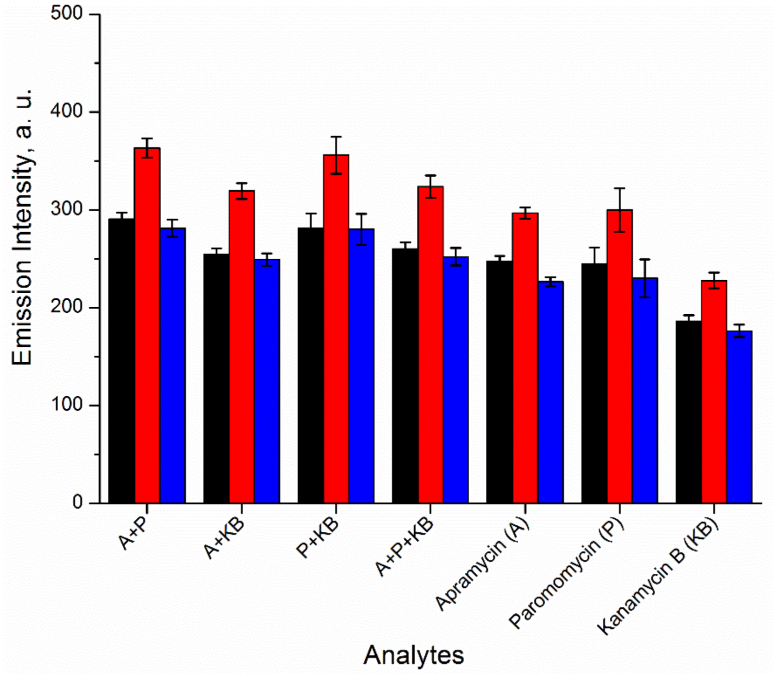


**Figure S29.** Fluorescence emission intensities ($\lambda_{ex}$: 335 nm) at 375 nm (black), 390 nm (red), and 410 nm (blue) of solutions containing dye **1** ([**1**] = 4 µM) and the analytes apramycin (A), paromomycin (P), kanamycin B (KB), or equimolar mixtures of these aminoglycosides ([analyte]$_{total}$ = 10 µM; 10 mM MOPS, pH 7.0). The values represent averages of five independent measurments.

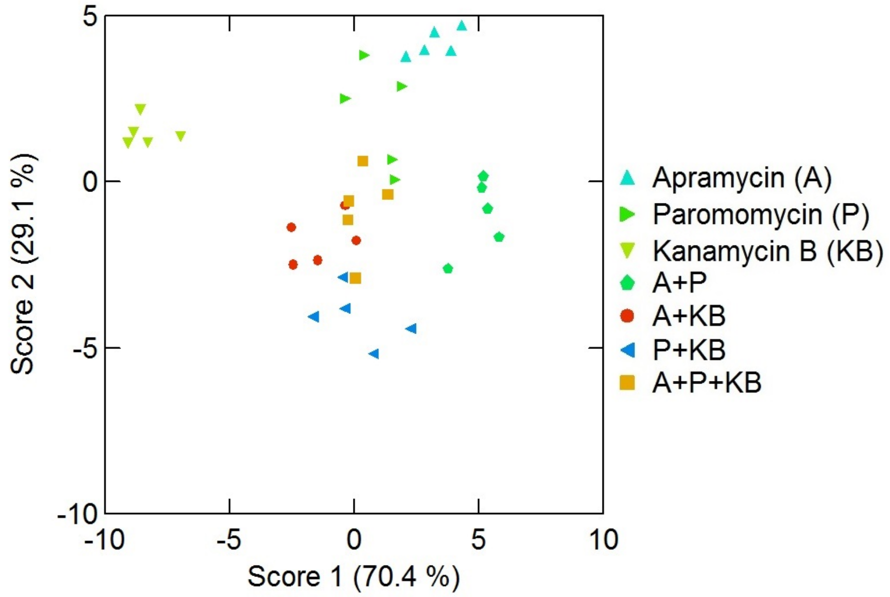


**Figure S30.** Two-dimensional LDA score plot for the analysis of apramycin (A), paromomycin (P), kanamycin B (KB), and equimolar mixtures of these aminoglycosides. The data were obtained as described above using only dye **1**.

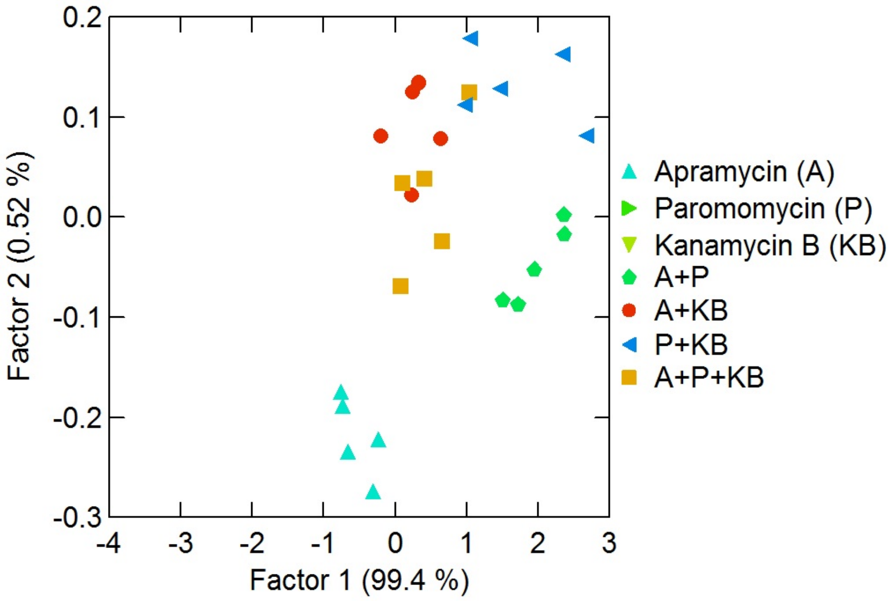


**Figure S31.** Two-dimensional PCA score plot for the analysis of apramycin (A), paromomycin (P), kanamycin B (KB), and equimolar mixtures of these aminoglycosides. The data were obtained as described above using only dye **1**.

### Sensing studies with dye 2 alone

The solutions were prepared as described above. A stepwise variable selection algorithm was employed to select three wavelengths: 380, 515, and 545 nm. The emission values at these wavelengths were used as input for linear discriminant analyses (LDA) and principal component analyses (PCA).

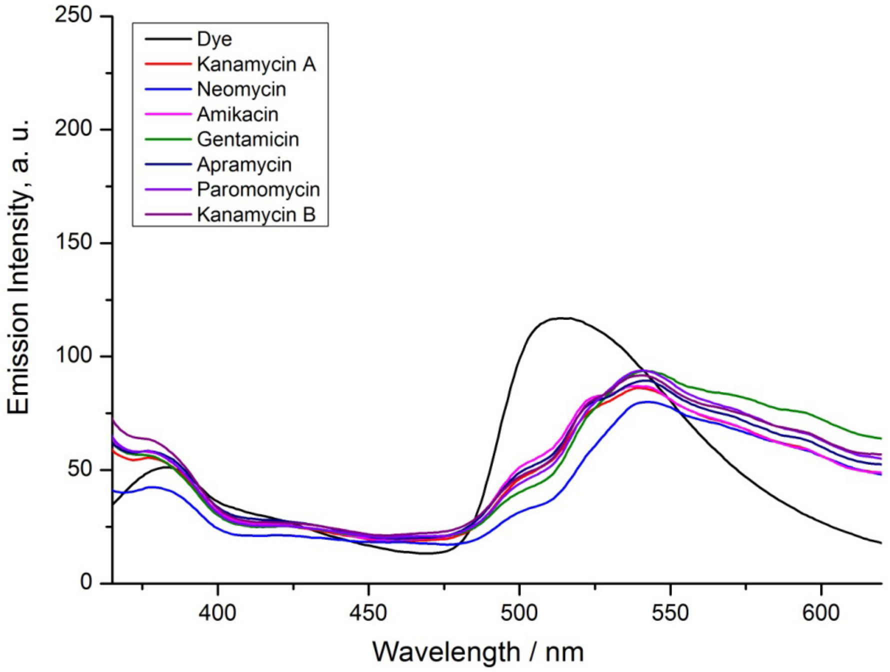


**Figure S32.** Fluorescence emission spectra ($\lambda_{ex}$: 335 nm) of solutions containing dye **2** (30 µM) and the analytes paromomycin, kanamycin B, kanamycin A, amikacin, neomycin, gentamicin, or apramycin ([analyte] = 10 µM; 10 mM MOPS, pH 7.0)

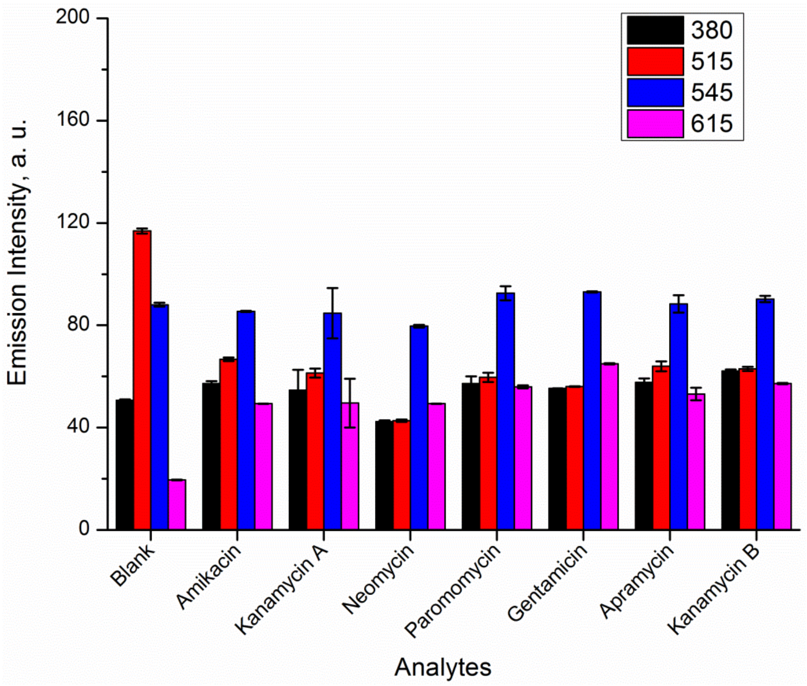


**Figure S33.** Fluorescence emission intensities ($\lambda_{ex}$: 335 nm) at 380 nm (black), 515 nm (red), 545 nm (blue), and 615 nm (pink) of solutions containing dye **2** (30 µM) and the analytes paromomycin, kanamycin B, kanamycin A, amikacin, neomycin, gentamicin, or apramycin ([analyte] = 10 µM; 10 mM MOPS, pH 7.0). The values represent averages of five independent measurments.

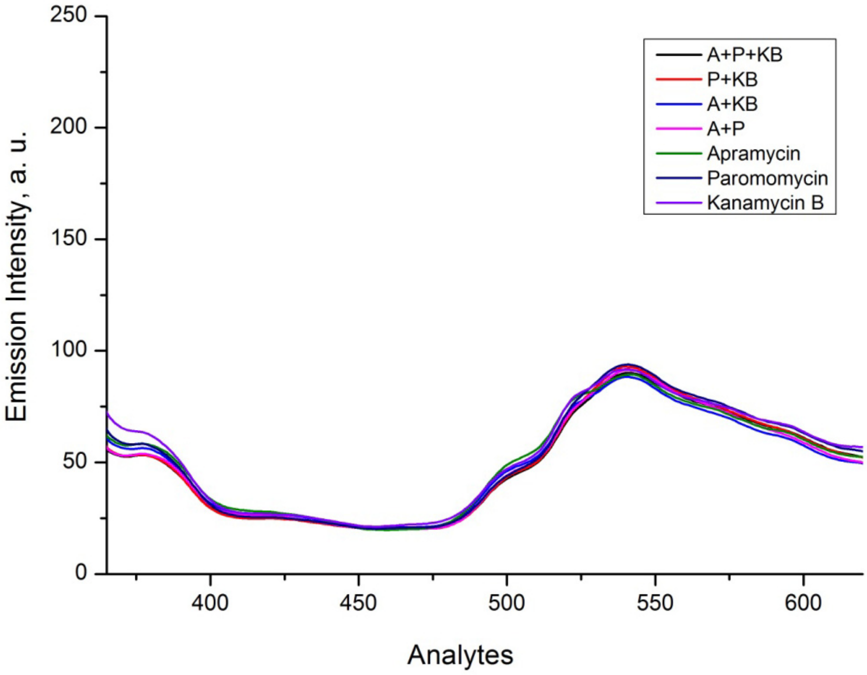


**Figure S34.** Fluorescence emission spectra ($\lambda_{ex}$: 335 nm) of solutions containing dye **2** (30 µM) and the analytes apramycin (A), paromomycin (P), kanamycin B (KB), or equimolar mixtures of these aminoglycosides ($[analyte]_{total}$ = 10 µM; 10 mM MOPS, pH 7.0).

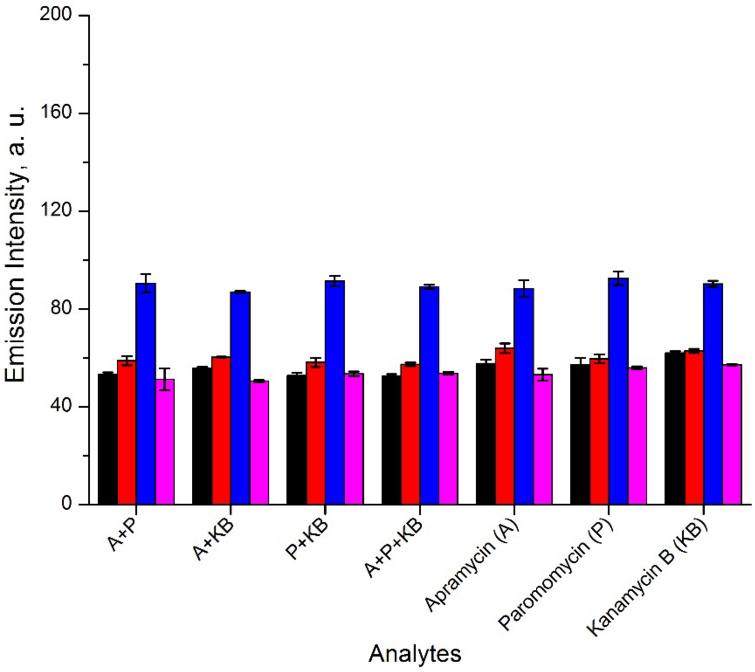


**Figure S35.** Fluorescence emission intensities ($\lambda_{ex}$: 335 nm) at 380 nm (black), 515 nm (red), 545 nm (blue), and 615 nm (pink) of solutions containing dye **2** (30 µM) and the analytes apramycin (A), paromomycin (P), kanamycin B (KB), or equimolar mixtures of these aminoglycosides ([analyte]$_{total}$ = 10 µM; 10 mM MOPS, pH 7.0). The values represent averages of five independent measurments.

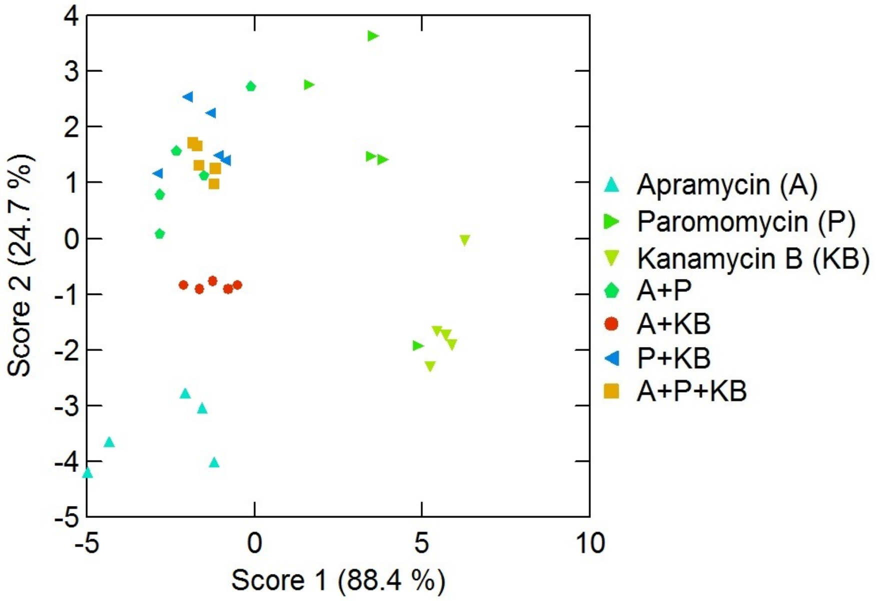


**Figure S36.** Two-dimensional LDA score plot for the analysis of apramycin (A), paromomycin (P), kanamycin B (KB), and equimolar mixtures of these aminoglycosides. The data were obtained as described above using only dye **2**.

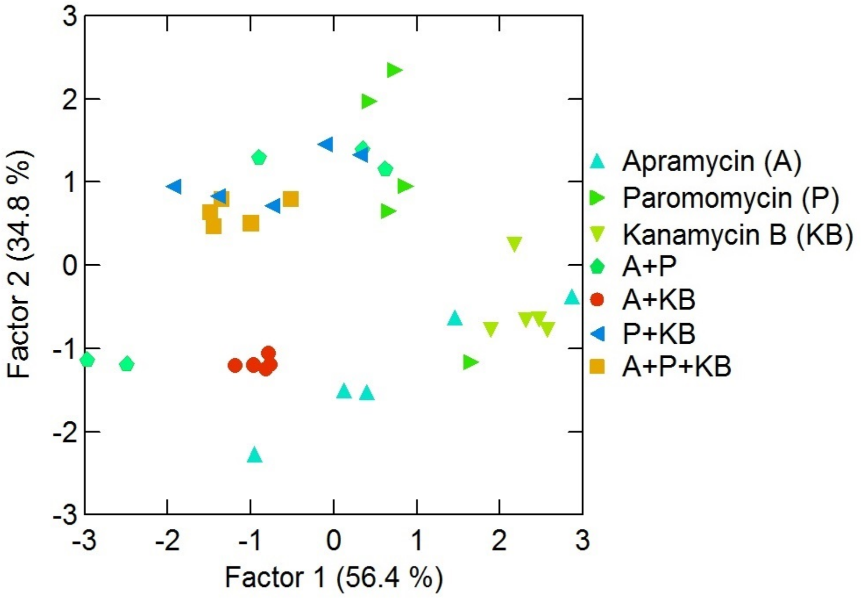


**Figure S37.** Two-dimensional PCA score plot for the analysis of apramycin (A), paromomycin (P), kanamycin B (KB), and equimolar mixtures of these aminoglycosides. The data were obtained as described above using only dye **2**.

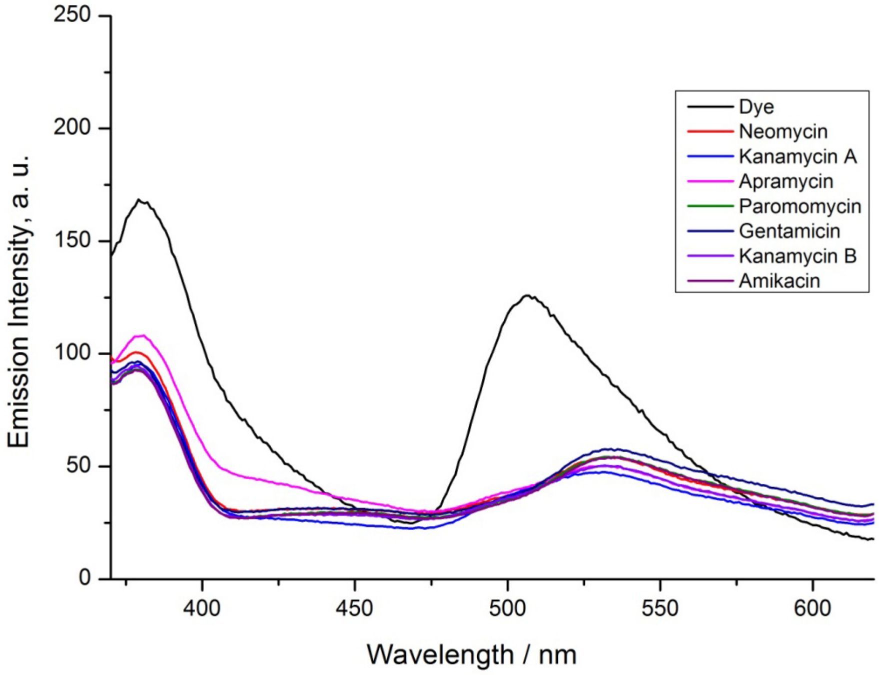


**Figure S38.** Fluorescence emission spectra ($\lambda_{ex}$: 335 nm) of solutions containing dye **2** (5 μM) and the analytes paromomycin, kanamycin B, kanamycin A, amikacin, neomycin, gentamicin, or apramycin ([analyte] = 10 μM; 10 mM MOPS, pH 7.0)

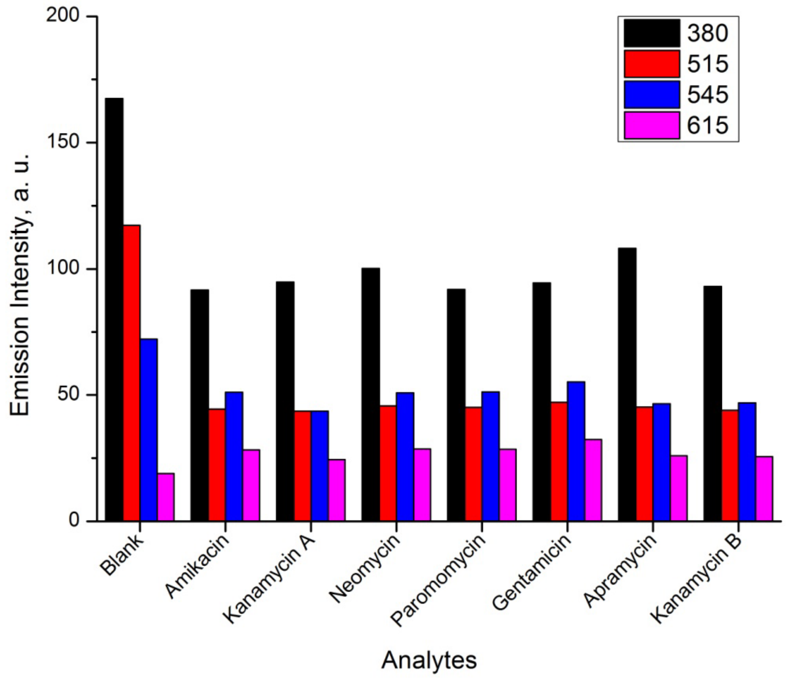


**Figure S39.** Fluorescence emission intensities ($\lambda_{ex}$: 335 nm) at 380 nm (black), 515 nm (red), 545 nm (blue), and 615 nm (pink) of solutions containing dye **2** (5 µM) and the analytes paromomycin, kanamycin B, kanamycin A, amikacin, neomycin, gentamicin, or apramycin ([analyte] = 10 µM; 10 mM MOPS, pH 7.0).

## 8. References:


1 H.-M. Kuo, S.-Y. Li, H.-S. Sheu and C. K. Lai, *Tetrahedron.*, 2012, **68**, 7331–7337.

2 L. Chen, H.-Y. Zhang and Y. Liu, *J. Org. Chem.*, 2012, **77**, 9766−9773.

3 M. Shah, K. Thangaraj, M.-L. Soong, L. T. Wolford, J. H. Boyer, I. R. Politzer and T. G. Pavlopoulos, *Heteroatom Chem.* 1990, **1**, 389−399.

4 A. Technologies, CrysAlis PRO, 2009−2014, Agilent Technologies Ltd, Yarton, Oxfordshire, UK.

5 G. Sheldrick, *Acta Cryst.*, 2008, **A64**, 112−122.

6 C. B. Minkenberg, F. Li, P. van Rijn, L. Florusse, J. Boekhoven, M. C. A. Stuart, G. J. M. Koper, R. Eelkema and J. H. van Esch, *Angew. Chem. Int. Ed.*, 2011, **50**, 3421–3424.
